# The History of Mental Health Services in Modern England: Practitioner Memories and the Direction of Future Research

**DOI:** 10.1017/mdh.2015.48

**Published:** 2015-10

**Authors:** John Turner, Rhodri Hayward, Katherine Angel, Bill Fulford, John Hall, Chris Millard, Mathew Thomson

**Affiliations:** 1 Centre for Health Care Management and Policy, University of Surrey, Guildford GU2 7XH, UK; 2 School of History, Queen Mary University of London, Mile End Road, London E1 4NS, UK; 3 Centre for the History of Emotions, Queen Mary University of London, Mile End Road, London E1 4NS, UK; 4 Faculty of Philosophy, University of Oxford, Radcliffe Humanities, Woodstock Road, Oxford OX2 6GG, UK; 5 Centre for Medical Humanities, Oxford Brookes University, Oxford OX3 0BP, UK; 6 Department of History, University of Warwick, Coventry CV4 7AL, UK

**Keywords:** Mental health policy, History of psychiatry, Service users, Risk, National Health Service, Decarceration

## Abstract

Writing the recent history of mental health services requires a conscious departure from the historiographical tropes of the nineteenth and twentieth centuries which have emphasised the experience of those identified (and legally defined) as lunatics and the social, cultural, political, medical and institutional context of their treatment. A historical narrative structured around rights (to health and liberty) is now complicated by the rise of new organising categories such as ‘costs’, ‘risks’, ‘needs’ and ‘values’. This paper, drawing on insights from a series of witness seminars attended by historians, clinicians and policymakers, proposes a programme of research to place modern mental health services in England and Wales in a richer historical context. Historians should recognise the fragmentation of the concepts of mental illness and mental health need, acknowledge the relationship between critiques of psychiatry and developments in other intellectual spheres, place the experience of the service user in the context of wider socio-economic and political change, understand the impacts of the social perception of ‘risk’ and of moral panic on mental health policy, relate the politics of mental health policy and resources to the general determinants of institutional change in British central and local government, and explore the sociological and institutional complexity of the evolving mental health professions and their relationships with each other and with their clients. While this is no small challenge, it is perhaps the only way to avoid the perpetuation of ‘single-issue mythologies’ in describing and accounting for change.

The history of psychiatry in post-war Britain has largely been told through two interlinked narratives: the rise of psychopharmacology and the process of ‘decarceration’. As Hess and Majerus noted in 2011,^1^ such narratives share with manifold histories of nineteenth-century psychiatry a concern with the issues of rights, confinement, treatments and the level of the asylum population. While these tropes have illuminated many aspects of the history of psychiatry, there is a striking divergence between the ‘single-issue mythologies’^2^ developed in these works and the sheer diversity of approaches to understanding and managing mental distress and disorder that characterises the British mental health services at the beginning of the twenty-first century. The scope and rapidity of change has left many developments in social policy, legislation, medico-legal practice, service design, service delivery and clinical practice without systematic historical analysis. New emphases in service provision, such as person-centred care, well-being, recovery, the involvement of service users and increased access to psychological therapies, lack a historical context. Indeed, the language of mental health has changed. A historical narrative structured around rights (the right to health and the right to liberty) is now complicated by the rise of new organising categories such as ‘costs’, ‘risks’, ‘needs’, ‘inclusion’ and ‘equality’, which contemporary actors use to define competing visions of mental health services. As a first step in tracing out the new language and landscape of mental health care, this paper sets out a research prospectus in the form of a report on a series of witness seminars and interviews concerning the history of mental health services since the Mental Health Act of 1959, which replaced the legislation under which services had been provided since the 1890 Lunacy Act. Seminars were held at the Wellcome Unit for the History of Medicine in London in 2010 and 2011, and were supported by the Wellcome Trust.^3^

## 1 Method

The explicit purpose of the seminars was to develop historical questions rather than to generate replicable answers. This had implications for the selection of speakers, the conduct of the meetings and the construction of the report which follows. The principal contributors were fifteen speakers: practitioners, policymakers, historians, service users and social scientists with diverse backgrounds and professional roles. These included two civil servants, three clinical psychologists, a psychotherapist, five psychiatrists, three social scientists of diverse specialisations and a third sector provider.^4^ In view of the purpose of the seminars, the group was not constructed as a representative sample. Some of the contributors were invited because they had played key roles in policymaking or debate in the recent or more remote past; willingness and availability played a large part in the final roster. It would in any case be difficult to embody a ‘typical’ range of clinicians in a few members of each profession and, indeed, any such selection on our part would have involved begging the question. While the absence of a biologically inclined psychiatrist, from a roster in which psychiatry was, if anything, over-represented, was perhaps the most obvious lacuna in the representation of professionals, there were many other varieties of psychiatry and psychology, and particularly of nursing and social work, which could only be represented by proxy in the accounts of the professional contributors. Many of the speakers had overlapping roles. Of the seven primarily involved in policymaking, six had also been practitioners; two of the psychiatrists and two of the clinical psychologists had extensive policymaking and management experience; three practitioners were also historians. One witness and a number of the other participants had experience as service users.

Each seminar was conceived to explore where possible the perspective of a particular group or profession. The first was devoted to service users; subsequent seminars addressed the views of psychiatrists, psychologists and policymakers, with further sessions addressed by single individuals. Seminars were also attended by a group of interested academics and postgraduate students, who contributed to the discussion. A number of speakers and many of the other participants attended for more than one seminar, lending a degree of continuity and integrity to the discussions in which service users and professionals were engaged.

Speakers were initially asked to act as witnesses rather than historians, reflecting upon their own experiences with a view to elucidating important themes in the history of mental health services in the period. Each seminar began with brief and relatively informal presentations, followed by questions and discussion. All the proceedings were taped and transcribed to provide the basis for this report. However, to encourage freedom of expression, speakers contributed under modified Chatham House rules, in that explicit permission would be required for any comment to be published.

Like many participants in élite oral history, contributors found it difficult to separate the roles of witness and historian. For the purposes of this project, this common pitfall was less of a problem than it would have been if we had been trying to use the material to create or validate an historical narrative.^5^ Many of the contributors had been engaged in practice and debate during the events which they were recollecting. Memories were well rehearsed, and framed by the language and assumptions of those debates. On certain sequences of events, they were sometimes demonstrably wrong. On certain subjects, nonetheless, it could be seen that participants were authentically reporting past perceptions. There was a striking congruence, for example, between the tone adopted by some contributors in discussing the early years of community-based services and the anxious, slightly sceptical foreboding which appeared in contemporary published accounts.^6^ This might be taken as corroboration of their interpretations, or as an example of the tendency of elite witnesses to create a story and stick to it. In some instances, by contrast, it became clear that the experience of participants contributed only a very preliminary stage in the development of an important question. A case in point is the discussion of resource allocation, in which participants knew that there was an issue, because they had felt the consequences, but did not formulate the question or identify methodological problems in the way that historians must. In other cases, such as the exploration of the influence of anti-psychiatry, it was evident that speakers were joining an ongoing conversation rather than conveying a coherent account of a situation in the past, and in such cases we have had to refer to existing literature to clarify their argument (and ours). Our investigation revealed further problems with the language of description, littered as it is with terms of art such as ‘recovery’ and ‘community care’ which not only divide professionals from the general public but are also given varying meanings within the professional community.

In the original project for the seminars, service users were identified as stakeholders on an equal footing with practitioners and policymakers. This was easier said than done. The service user view was put forward in at least four ways: by service users who were also academics with an interest in the service user voice; by other service users in the audience; by proxy in the contributions of mental health professionals who were not service users; and by proxy in the contributions of academics who were interested in service user issues but who were not themselves service users. None of these modalities can be regarded as perfect. Necessarily, because service users as a category are even more heterogeneous than professionals or policymakers, no claim to typicality should be made for the service user contributors; some spoke as individuals, some spoke in a role defined wholly or partly as a service user role which would be construed as representative. Service users, however counted, were also hugely outnumbered by professionals of some ilk. As a result, although the service user voice emerges pervasively in the discussion, it does so rather more diffusely than the professional voices.

The material presented in the rest of this paper represents a distillation by the authors of the key concerns which emerged from the seminar discussions, informed by existing literature on mental health services in the period, which is referenced in footnotes. The contributions of the speakers are to a large extent privileged over the comments of the audience, which generally appear as background commentary without footnote reference. Through a series of consensus meetings in which the authors collectively reviewed the transcripts, we have selected for discussion those themes which were most salient to all three ‘stakeholder interests’ – practitioners, policymakers and service users – but note also, in the conclusion, other prominent themes which demand fuller historical exploration. The main discussion themes, interestingly, tended to recur across groups and were not confined to one session. Notwithstanding these caveats, we would contend that the seminar discussions provide the basis for a new programme of enquiry which would reflect the lived experience of participants and, *inter alia*, respond more fully to the service user perspective than more traditional accounts.

**Figure 1:**
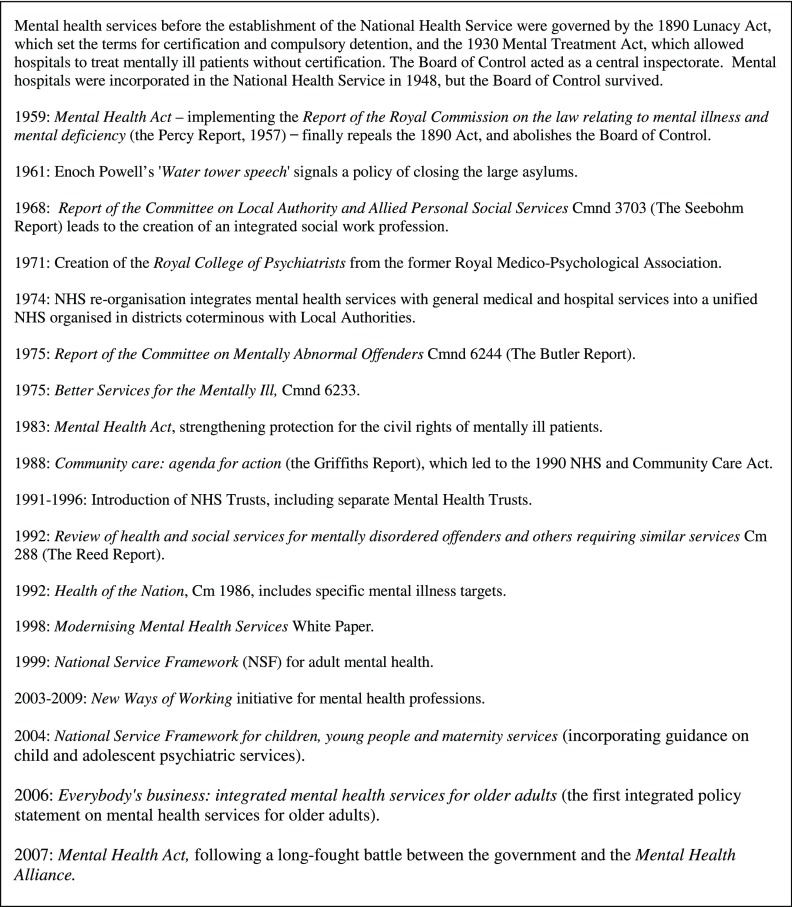
Some key dates in mental health policy since 1959.

## 2 The context

Figure 1 presents a timeline of key activity in mental health policy in the period covered by contributors’ careers. The 1959 Mental Health Act and the 1962 Hospital Plan presaged the rundown of the asylums and the assimilation of psychiatric care into the wider hospital system. Services thereafter developed against a background of serial reorganisations of the NHS and local government. In the 1970s both Labour and Conservative governments acknowledged, but did not address, the need to provide more resources to deliver mental health services in the community^7^ while evidence mounted of inadequate care within psychiatric and mental handicap hospitals.^8^ After 1979, the new Conservative government first reorganised the administration of the NHS in 1984, imposing general management embodied in a new structure of health authorities. It then reorganised the whole service again through the 1990 National Health and Community Care Act, which laid the principal responsibility for community-based care on local authorities. Until 1997 the improvement of care for people with severe mental illness was the principal focus of policy. This was marked by a preoccupation with ‘dangerous’ people as much as with the provision of resources.^9^ Throughout the period, public spending on mental health was low compared with physical health, particularly with regard to services for children, adolescents and the elderly.

The 1997–2010 Labour governments increased spending on mental health, though not by as much as the general increase in NHS expenditure. The plan was laid out in *Modernising Mental Health Services* in 1998 and executed as part of target-driven reforms of NHS provision. The 1999 National Service Framework (NSF) for Mental Health set out specific objectives, though only for adults of working age. Following this, mental health was one of three declared clinical priorities alongside cancer and heart disease, in the 2000 *NHS Plan*. Targets were accompanied by promises of funds and an unprecedented level of detailed guidance from the Department of Health. In 2006 the *Improving Access to Psychological Therapies* (IAPT) programme was introduced with the explicit rationale of reducing the economic burden to the country of mild to moderate mental illness. Labour also sought to extend and clarify the powers of compulsion over mentally ill patients which had been codified in the 1959 Act and mitigated substantially in the 1983 Mental Health Act. The new Mental Health Act was finally passed in 2007. The Labour government’s programme dominated the recent working lives of the seminar contributors. The coalition government’s policy paper, *No Health without Mental Health*, was only published in February 2011, while the seminar programme was under way.

Changes over the period can also be described, if only partially, in figures. Table 1 illustrates some key aspects of change. In the broadest of terms, the mental health service in 1950 compulsorily detained most of its users for long periods in large asylums, attended by nurses under the supervision of doctors. Sixty years later, most service users passed most of their lives without legal constraint outside hospital, supported by a number of professions. The range of problems addressed by the services was much larger, and the expectation of cure or relief of symptoms was much higher. That said, those with severe mental illness were still liable to spend time in and out of hospitals (as demonstrated by the changing ratio of admissions to occupied beds) and may indeed have been spending longer in total than similar patients as much as a century before.^10^ Since the 1990s, the number of those compulsorily detained has begun to grow again. The table does not show the provision of services by primary care or by local authority social service departments, which took increasing responsibility for residential care and some community-based care across the period. Nor does it show the growth of provision outside the NHS, in the form of private practice by psychiatrists, psychologists and counsellors, the provision of services by user groups and charities, and the growth of private hospitals offering services directly as well as by contract to the NHS.

**Table 1:** Secondary mental health services: NHS in England and Wales selected summary data.

	**1950**	**1960**	**1970**	**1980**	**1990/01**	**2000/01**	**2010/11**
**Patients**							
*Mental health in-patients* ^i^ *’000s*	147.3	140.6	106.4	79.6	*49* ^ii^	32.1	22.7
*Average length of stay (days)* ^iii^	*863*	*355*	218	*149*	*83*	65	61
*Total inpatient admissions ’000s*	60.2	N/R	166.0	192.0	237.9	189.9	136.5
*Of which compulsory admissions*	19.5		29.4	N/R	19.8	23.1	31.8
*Patients compulsorily detained at year end ’000s’* ^iv^	120.3	17.2	7.5	N/R	14.1	13.8^v^	16.6^vi^
*Learning disabilities in-patients ’000s* ^vii^	51.3	57.2	54.4	46.3	*21* ^viii^	5.5	1.7
*New out-patients (MH & LD)* ^ix^ *’000s*	102.8	167.4	217.0	198.6	227.4	304.6	413.9
*Out-patient attendances (MH & LD) ’000s*	523	1265	1531	1691	1830	2176	2642
**Workforce**							
*Consultant psychiatrists*	454	679	1054	1607	2116	3057	4850
*Of which mental health*	*N/R*	*N/R*	*942*	*1455*	*1933*	*2855*	*4545*
*Of which learning difficulties*	*N/R*	*N/R*	*112*	*152*	*183*	*202*	*305*
*Mental health nurses ’000s* ^x^	17.5	25.0	28.7	36.1^xi^	37.0^xii^	44.6	49.0
*Learning disability Nurses ’000s*	5.5	9.8	14.5			10.6	6.5
*Clinical psychologists* ^xiii^	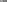	179 wte	399 wte	1078 wte	2200	5316	8837
*Psychotherapists*				80 wte	169	663	2025
*Population of England and Wales (million)*	43.7	46.1	49.1	49.6	50.5	52.1	55.2

i Average daily occupied beds.

ii Average occupancy not published for this year. Figure estimated from published available bed figures, using 1986 occupancy ratio.

iii Figures in roman type drawn directly from published reports. Figures in italic calculated from published figures by dividing the number of discharges into the average bed occupancy multiplied by the number of days in the year. The dramatic drop over the period in average length of stay was caused to a significant extent by the non-replacement of long-stay patients who died in hospital, or were discharged into the community in the 1990s. In 1954 the Percy Commission estimated that 46% of patients in mental hospitals had been resident for more than ten years, and 10% for more than 30 years. About 40% of new admissions in the 1950s were discharged within three months and 80% within a year; by 2010/11 these figures had risen to 80% and 95%.

iv Includes patients confined in special hospitals and patients detained because of ‘mental impairment’.

v England only: Welsh figures not recorded.

vi England only. In addition, 4.5 thousand patients outside hospital were subject to compulsory treatment orders.

vii Changes in this statistic reflect movement from NHS to local authority and private residential provision. No consistent series of data exist for those outside NHS residential care. A crude indicator of the overall growth in the number of people with learning disabilities receiving statutory services (and thus served by the professionals enumerated in this table) is given by the comparison between the sum of inpatients, persons ‘under guardianship’ and persons ‘under statutory supervision’ in 1950 (c. 109 thousand) with the 189 thousand adults ‘known to services’ and 286 thousand children recognised as having learning disabilities estimated (for England alone) for 2011 by Emerson *et al.* *People with Learning Disabilities in England 2011: Services and Supports* (London: Learning Disabilities Observatory, 2012).

viii Estimated from availability figures and 1986 occupancy ratio.

ix Outpatient activity for learning disabilities was a tiny minority of all outpatient activity.

x Headcounts, including all non-student nurses. In 1970 almost a quarter of non-student nurses had no qualifications. Figures from 1990 report only qualified nurses.

xi Figures for mental health and learning difficulties reported without differentiation in these years. Data for England only: using the ratios of 2000, the Welsh complement would be between 4 and 7 thousand.

xii Figures for mental health and learning difficulties reported without differentiation in these years. Data for England only: using the ratios of 2000, the Welsh complement would be between 4 and 7 thousand.

xiii Numbers for clinical psychologists were only reported as whole-time equivalents until 1980. From 1990, numbers included only qualified psychologists.

*Notes*: 1. Data are drawn from the following publications. Data from England and Wales were aggregated together in official publications before 1974. a. 1950: *Report of the Ministry of Health covering the period 1st April 1950 to 31st December 1951*, Cmnd 8655. b. 1960: *Report of the Ministry of Health for the year ending 31st December 1960; Part I, The Health and Welfare Services* Cmnd 1418; *Report of the Ministry of Health for the year 1961. Part II, On the state of the public health. Being the annual report of the Chief Medical Officer*, Cmnd 1856. c. 1970: *Department of Health and Social Security Annual Report 1970*, Cmnd 4714. d. 1980: Department of Health and Social Security, *Health and Personal Social Services Statistics*, 1982 & 1983 edns (London: HMSO); Welsh Office, *Health and Personal Social Services Statistics for Wales* 1982 &1983 edns (Cardiff: HMSO). e. 1990: Department of Health and Social Security, *Health and Personal Social Services Statistics*, 1992 & 1993 edns (London: HMSO); Welsh Office, *Health and Personal Social Services Statistics for Wales* 1992 & 1993 edns (Cardiff: HMSO). f. 2000 & 2010: English data as follows: Patient data from http://webarchive.nationalarchives.gov.uk/20041108195217/http://www.performance.doh.gov.uk/ (2000/01), http://www.ic.nhs.uk/hes and http://data.gov.uk/dataset/mentalhealth-bulletin-fifth-report-from-mental-health-minimum-dataset-mhmds-annual-returns-2011 (2010/11); Formal detention data for 2000/01 from Department of Health, *In-patients formally detained in hospitals under the Mental Health Act 1983 and other legislation, England: 1990–1991 to 2000-2001* (London: 2001); Workforce data from https://catalogue.ic.nhs.uk/publications/workforce/numbers/nhs-staf-medi-dent-1995-2005/nhs-staf-medi-dent-1995-2005-rep2.pdf (2000/01); http://www.data.gov.uk/dataset/nhs-staff-2000-2010-medical-and-dental (2010/11). Welsh patient and workforce data all from https://statswales.wales.gov.uk/Catalogue/Health-and-Social-Care. 2. N/R indicates that official data for an element were not recorded or published on a national basis. 3. The data are only as good as contemporary methods of collection and aggregation. Increases in the complexity of data collected across the NHS in recent decades, accompanied by a growth in the number and type of reporting entities, have improved the validity of some measures but reduced the reliability of national aggregates. Increasing use of data for performance management has increased the probability of manipulation both at the point of collection (at the patient interface) and at the point of reporting (Strategic Health Authorities or Trusts), and thus reduced the reliability of aggregates; this uncertainty applies particularly to admissions, length of stay and outpatient activity in 2000 and 2010. Patient data and nursing workforce data are rounded, partly to reflect the possible inaccuracies and inconsistencies in official statistics. 4. Except where indicated, staff numbers are headcounts. Changing reporting conventions make it impossible to present whole-time equivalents on a consistent basis across the period.

The ‘patient journey’ into specialist mental health services also changed substantially. Until 1959, patients would have been referred by GPs to psychiatrists working in hospitals and certified, if needed, under the 1930 Mental Treatment Act. Their treatment would have been determined by the Physician Superintendent of the hospital. From then until 1983, the route from the GP would have been to a consultant psychiatrist at an outpatient clinic, or sometimes directly to a clinical psychologist; the clinical process would have been controlled by the consultant or the psychologist. Between 1983 and 2000, GPs would increasingly refer to multi-disciplinary Community Mental Health Teams, where other professionals, or GPs themselves, might assume responsibility for the clinical process.^11^ The turn to Community Mental Health Teams was underwritten by the Department of Health’s institution of the Care Programme Approach in 1990. Under this scheme, key workers from either the NHS or local authority social services would be charged with coordinating the delivery of individual patient care.^12^ Under the National Service Framework after 2000, GPs might refer to one of a number of different specialised teams. Under the *Increasing Access to Psychological Therapies* (IAPT) programme after 2006, people experiencing mental distress could also refer themselves to some services without GP intervention.^13^

For many, though, the patient journey never started, and for most it was very short. The prevalence of mental disorder not dealt with by specialist services became a topic of discussion in the 1960s.^14^ A WHO study in the early 1990s estimated that for every thousand adults, between 250 and 315 were suffering from some sort of mental disorder, of whom only 101 were detected by GPs, only 20.8 were referred to specialist mental health services (including community-based services) and only 3.4 became in-patients.^15^ In recent years the rate of referral per thousand adults has probably increased,^16^ but it remains the case that the majority of mild to moderate illness is treated by GPs, if at all.

## 3 History as retrospective: contributors’ reconstruction of their own past

At least for the earlier years a broad consensus emerged about the main characteristics of the mental health services. The 1960s were acknowledged as a period of slow but necessary change. Services were dominated institutionally and intellectually by psychiatrists, who began to establish a more distinct professional identity and formal training under the Royal College of Psychiatry (chartered in 1971). ‘When I was being trained in psychiatry we thought that we were the experts and we decided.’^17^ Senior psychiatrists tended to see the 1959 Act as benign in its impact on services and patient experience, partly because it allowed them, through the procedure of voluntary admission to mental hospitals, to implement improvements in treatment and care which had been foreshadowed in the 1950s. ‘…within limits, particularly financial limits, doctors could do what they thought was best. If somebody had a bright idea for a new service, they could do it, provided it didn’t cost very much.’^18^ The immediate impact on mental health services of Enoch Powell’s Hospital Plan was to achieve the union of psychiatry with medicine and create conditions for effective treatment in the community, while promising the eventual closure of the old asylums. For this reason, long-serving psychiatrists saw Enoch Powell as a progressive figure in mental health reform.^19^ At the same time, contributors acknowledged that psychiatric treatment (‘some sort of a mystic process’^20^) was barely supported by evidence and often bizarre (‘the eccentricities of some of the treatments used when I was a trainee simply would make your hair stand on end’^21^), and care was perceived by service users as ‘awful’.^22^

Dissatisfaction with the status quo, as recollected by contributors, was manifested in a number of ways. The intellectual challenges of ‘anti-psychiatry’ in the writings of David Cooper *et al.* coincided with a general counter-cultural challenge to medical authority.^23^ The therapeutic pessimism of the asylum system was challenged by hopes for new pharmacological interventions, while the lack of sufficient resources for community care of the mentally ill was already causing frustration to clinicians. The earliest service user movements appeared in the early 1970s, demanding civil and economic rights for patients in the community, and, in parallel, pressure groups such as MIND began to agitate for changes to the 1959 Act. Yet the 1970s also saw significant innovations in treatment and service delivery, led by clinicians responding to these challenges. There was increasing use of psychological treatments with an evidence base and widespread acceptance that the services needed to acknowledge and counteract the social devaluation of their users.^24^

From the mid-1970s, structural reorganisation and disruption figured more strongly in recollections, though it was still said that ‘in spite of Enoch Powell’s apocalyptic threats in 1962, the 1980s was the best time that the mental hospital ever had. Money wasn’t flowing freely but there was more than there had been; numbers (of patients) were falling, while the staff numbers were being preserved. So the standard of nursing care was going up. And there was, in most cases, enough to make the old accommodation certainly tolerable, if not quite satisfactory.’^25^ The major reorganisation of the NHS initiated by Keith Joseph in 1974 demanded a new strategy for delivering community-based care, and the subsequent Labour government, operating under great financial stringency, accepted the thrust of the reorganisation but increased the mental health budget by a mere 1.8% to deal with the consequent needs. Clinicians remembered this process as doubling the number of managers in the NHS,^26^ though some of these were undoubtedly existing senior clinicians rebadged as managers. More significant changes followed the first Griffiths Report in 1983 into the management of the NHS.^27^ This recommended the introduction of regional and district managers into the health service and the devolution of decision-making to hospital level. The second Griffiths Report of 1988^28^ and the 1990 National Health and Community Care Act were seen to further increase levels of managerialism, creating the purchaser/provider split in the NHS and the clear allocation of the principal responsibility for community-based care to local authorities. Griffiths also ‘promoted the use of the independent sector, as it was primly called at the time. This is private medicine of course.’^29^ Witnesses were manifestly aware that in this somewhat chaotic climate they themselves, as front-line workers, were significant in shaping, for better or worse, the public policies they were supposedly implementing, when community care was ‘a kind of shared myth’ without clear definition.^30^ This did not reduce the remembered frustration at the inadequate resources made available: ‘for a long time community care was largely a myth: everybody agreed it was a good thing and we should have more of it, but resources were simply not allocated in that direction.’^31^

Thus for the last two decades of the twentieth century a consensus memory was superseded by multiple, interlocked narratives. Professionals divided into negative and positive camps, with the division cutting across professional boundaries. The disenchanted professional view held that, as above, the movement of patients out of psychiatric hospitals into the community – ‘decarceration’ – was a good idea spoiled by inadequate resources. From this perspective, public policy was at fault, because ministers would not fund or direct community care but pandered to public prejudice by imposing greater restrictions on patients and professionals, especially in the 1990s and after, when politicians renewed their interest in mental health policy. Witnesses argued that a renewed emphasis on control and confinement was a bad policy and that it grew from a political reaction to scandal: specifically, homicides by psychiatric outpatients, such as the killing of Jonathan Zito by Christopher Clunis in 1992. This was held to have led to an emphasis on forensic services, with a diversion of resources from other aspects of mental health into High Dependency and Medium Secure Units, ‘the new lunatic asylums that Frank Dobson dreamt up’.^32^ Response to crisis was held to explain new organisational and procedural demands on mental health services such as the Care Programme Approach (‘a central imposition [in the] 1994 guidance arising out of the Clunis affair’^33^) and the development of specialist structures targeted largely at individuals deemed to be a risk to themselves or others. These included assertive outreach teams and crisis resolution and home treatment teams created under the 2000 NHS plan, which were associated by some clinicians with fragmentation of care.^34^

A more optimistic professional perspective saw a set of positive developments in the same period. On the one hand, closure of the long-stay hospitals was associated with significant advances in psychiatric rehabilitation, involving multi-disciplinary teams working in the community.^35^ This was associated with new professional aspirations, particularly for social workers, in a climate where ‘actually the mission was about helping people achieve an ordinary life, whatever that meant to them’.^36^ Practice was influenced by the advice of service users, often brought in to train professionals, and the role of the professional was increasingly that of an advocate for the service user. While a focus on the most needy was associated with the management of risk, the profile of mental health services was raised by the 1992 Health of the Nation white paper which ‘put mental health on the map in slightly sort of morbid terms because suicide was the target, and people at the time talked about that being a kind of proxy, a way of getting some attention to mental health.’^37^ Over the same period, evidence-based developments in psychological treatment expanded the range of interventions available, and, more generally, the development of a scientific literature led to a greater homogenisation of methods,^38^ though the disenchanted view was that drug treatment still predominated and that ‘despite the powerful research literature and the heavy artillery of guidance from the National Institute of Clinical Excellence’ [founded in 1999], psychosocial interventions had not penetrated into routine clinical practice.^39^

The same years furnished wholly different insights from and about the users of services. Notwithstanding innovation, goodwill and improvement on the part of providers, mental disorder for the service user continued to be associated with social exclusion and the denial of civil rights. Although the 1983 Mental Health Act brought in new procedures, in practice patients legally confined in mental hospitals were rarely successful in their challenges to the system, and although voluntary patients could make decisions about their treatment, ‘if you don’t say yes you’re likely to find yourself detained’.^40^ For the growing number of patients outside hospital, ‘the problem with community care is not just about management; it is about misery, poverty and the style of mental health services that offers no real choice about the type of support available. The kind of service provided by mental health professionals is not the only, or necessarily even the main, issue that determines the quality of people’s lives in the community.’^41^ There was growing awareness that service users’ experience was skewed by ethnicity and gender: young black men were disproportionately subject to compulsory treatment for severe mental illness, women were over-represented among users presenting with mild to moderate illness. The growth of the service user movement provided a forum in which these concerns and complaints could be articulated.

These three narratives, from disenchanted professionals, from optimistic professionals and from service users, formed the background to discussion of the development of policy and services under the Labour government, which (as previously noted) dominated contributors’ recollections and concerns. Because, as one contributor remarked, changes in the recent period were, arguably for the first time, ‘a led process’,^42^ a fourth, policymakers’, narrative emerged. From this viewpoint, the Labour government offered a particular moment of political clarity, a model for implementation built on a target culture, a belief (not shared by previous administrations) in the value of national leadership on social policy issues, and above all the availability of money, all of which led to large positive changes. There was also a desire to modernise mental health legislation by superseding the 1983 Act. Clinicians in the group were sceptical of the actual effect of the target culture on outcomes, reckoning that the unprecedented detail of the Policy Implementation Guidelines (the Department of Health’s detailed instructions to NHS organisations^43^) militated against the provision of locally appropriate services. The opposite case was put by those working in policy development who argued that new strategies needed to be monitored and measured because ‘the NHS…is not quite a black hole, but it is a complex policy-eating environment’.^44^ Where clinicians saw a reduction in their influence, department officials pointed to the large constituencies of professionals consulted in the external reference groups for the National Service Framework even though civil servants rather than the reference groups actually wrote the policy.^45^ Similarly, service users and officials had different views of the reality of service user involvement in policymaking and of the ‘user-friendliness’ of policies, with the suggestion that the service user movement was sometimes ‘hijacked’ by clinicians and civil servants.^46^ The proposition that New Labour’s policy for mental health was unprecedentedly evidence-based was challenged in a number of ways. Policy innovation was traced to leaps of faith and changing values as well as to the evidence base. Yet it was argued that some policies held up as politically driven were in fact built upon earlier clinical arguments and experimental evidence: for example, Lord Layard’s IAPT programme built upon evidence that untreated anxiety and depression placed a large burden on primary care services and that patients preferred non-pharmacological therapies, but laid a new emphasis on the economic case that high levels of mild to moderate mental illness in the population were a drag on prosperity.^47^

Witnesses acknowledged that New Labour delivered large expenditure in mental health, but contested the effectiveness of that expenditure. For many user groups, notably black and minority ethnic groups, it was argued that there had been little change for the better despite broadly good intentions.^48^ Moreover, the increase in expenditure on targeted activities had been accompanied by disinvestment in other areas, and a very large proportion of new spending had been directed at secure environments and forensic services rather than at the community services which might reduce the need for such services.^49^ Services continued to vary significantly across the country. For clinicians, the development of clinical governance, which by implication made clinicians accountable to managers, and thus ultimately to the state and to the community at large, as well as to their patients, was a significant change. Alongside the recognition of extra expenditure on services was the view that New Labour had been socially authoritarian^50^ and used ‘Orwellian’ institutions to deal with personality disorder.^51^ Service users and sceptical practitioners alike saw the 2007 Act as a means of social control with little benefit to the welfare of service users.

Strikingly, the different narratives of recent developments in mental health services describe the fragmentation of a system which was regarded (arguably wrongly) as relatively homogeneous in an earlier period.^52^ The very definitions of mental distress and disorder had been widened, and thus the scope of services had been increased. Responsibility for providing these services, which had previously rested with the NHS and local authorities, had been extended to include the third sector and, increasingly, profit-making contractors. Psychiatric hegemony was challenged by new professions, by new conceptions of mental distress, and by a new assertiveness on the part of service users, as well as by the incursions of public policy and policymakers into clinical autonomy.

## 4 Signposts for historians

The four voices in these discussions – professionals (both approving and disenchanted), service users and policymakers – shared a number of common preoccupations which we argue should be central to future study of recent mental health services in England and probably elsewhere in the United Kingdom. The most important of these were the rise of the service user, the risk agenda, the allocation of resources, changing and contested definitions of mental health and psychiatric need, and the impact of changing professional values on the delivery of services. Any articulated narrative of change since 1959 must be informed by these concerns.

### 4.1 Service users

There was consensus among witnesses that one of the most important and striking changes in the history of post-war British mental health care has been the rise of the service user perspective. Forms of organised advocacy for users of mental health services have existed in Britain since the formation of the Alleged Lunatics’ Friend Society in 1845.^53^ However, the standard account of the emergence of the modern service user movement starts in the 1970s with the founding of groups that included the Mental Patients Union (founded in 1972) and then, when this broke up, organisations including the Community Organization for Psychiatric Emergencies (COPE), Protection for the Rights of Mental Patients in Treatment (PROMPT) and the Campaign Against Psychiatric Oppression (CAPO) which connected the movement to developments in the 1980s.^54^ The small scale and transient nature of many of the service user groups has made accurate assessment of the scale of the movement difficult, but the leading authority on the subject suggests that it expanded from about a dozen groups in 1985 to over 500 by 2005.^55^

The challenge for historians is to explicate the links between these developments and, on the one hand, the scientific and cultural critiques of psychiatric practice covered by the term ‘anti-psychiatry’ (and discussed further below) and on the other hand the internalisation of service user demands into the actual practice of mental health services towards the end of the period. Witnesses tended to echo Crossley’s view that the early movements owed as much to social radicalism as to the intellectual anti-psychiatry of the 1970s, though service users in the seminars challenged the philosophical basis of diagnostic categories and treatment methods in terms which would have been familiar in those earlier debates. Discussion of the later period was much less definite. The optimistic professional view of developments since the 1980s was that the civilising influence of campaigning organisations such as MIND and other groups had successfully engaged professionals in collaboration with service users in the design and delivery of the services, and indeed that this was a relatively unusual and advanced aspect of British practice.

This generous and present-centred^56^ perspective was contested from a number of directions. Contributors, including departmental officials, noted the lack of progress in addressing health inequalities in general, and in particular the disproportionately bad experience of service users from black and minority ethnic groups. The service user objections to the restrictions of liberty contained in mental health legislation are clearly alive and well after the 2007 Act, alongside a more consumerist critique of the inadequacy of resources to meet treatment needs. Nor have professionals and service users reached an easy consensus on treatment methods or service design. Research led by service users on electro-convulsive therapy, for example, met with considerable hostility from the Royal College of Psychiatrists before being acknowledged in the NICE guidelines on risk and consent. ^57^ Service users in the seminars also challenged the ethical basis of the ‘screen and intervene’ approach which might prompt services to subject too many people, too early, to active treatment for mental illness and argued that the emphasis on work and social inclusion in the recovery movement (a concept developed within the service user community) could constitute a threat of social control as much as a response to individual needs, echoing Joel Braslow’s critique of ‘recovery’ as nesting ‘neatly within the broader context of neo-liberalism’.^58^ Greater exposure for the service user viewpoint has manifestly not been followed by complete satisfaction of service user demands, and this clearly requires nuanced historical treatment.

### 4.2 Risk

A consistent theme in discussion was the distorting influence of a focus on risk, and the perception that this was a characteristic of recent policy and legislation. While contributions tended to be grounded in the specific ‘risk agenda’ of clinical and management practices within the mental health services and their significance for the social control function of psychiatry, some participants were clearly aware of the larger theoretical perspectives on risk raised by Ulrich Beck, Robert Castel and Niklas Luhmann and extended by Nikolas Rose for mental health.^59^ As one witness argued, ‘in terms of being admitted to psychiatric hospitals, and particularly under compulsion, there is an issue of risk, so it’s not just diagnosis that gets you admitted, it’s that you’re “risky”. And the big risk that all the psychiatrists worry about is violence.’^60^ The ‘risk of violence’ – perpetrated by the mentally disordered on members of the public – emerged as paramount in the circumscription of psychiatric patients’ freedom. But the political salience of risk was recognised as being neither new nor confined to mental health. Psychiatrists acknowledged that

…these waves of control and security, of course, don’t just touch mental health services, they touch the whole educational system, they touch the way people are allowed to take risks at other things. You know the old “’ealth and safety” industry is around…I think that wave went with the wave of “we must protect the members of the public from these raving lunatics at all costs”.^61^

The effect has been that ‘risk avoidance has been seen as a key public function of psychiatry …the current policy on mentally disordered offenders is almost wholly to do with public protection and not much to do with humanitarian concerns for the welfare of the individual.’^62^

However, mental health/violence scandals have been around for much longer than the Clunis affair which ostensibly prompted the repressive aspects of recent policy and legislation. The now largely forgotten case of outpatient Ronald Derek Sowle, who killed an eighteen-year-old Bristol schoolgirl in 1961 while living in a psychiatric hostel, three days after having been released from detention under the Mental Health Act and reclassified as an informal patient, caused a series of questions to be asked in Parliament and a full enquiry by the local authority concerned but did not come anywhere near derailing the ‘community care’ or mass decertification processes arising from the Mental Health Act 1959.^63^ For many years thereafter the ‘problem’ of dangerous offenders was subsumed in a nuanced discussion of the best way to promote co-operation between the justice system and the mental health services, an approach epitomised in the 1975 Butler report, which, among many other things, launched the development of forensic psychiatric services in the NHS.^64^ It would seem that it is not the content of the ‘scandals’ that determined policy changes, but the broader traction granted by the rise of ‘risk’ as a discourse in the public sphere.

In the seminars, the risk agenda excited passions of two sorts. Clinicians tended to interpret it as an excuse for inappropriate bureaucratic interference in their clinical judgement:

…the one thing that needs to be understood out of all that stuff about danger and risk, which is monstering us, is that because of the impositions of risk assessment and risk management, the forensic establishment and so forth, we’re now in danger of neglecting the non-dangerous patient.^65^

This echoes the views of Rose and Castel, who see the risk agenda as diminishing the standing of psychiatrists.^66^ Clinicians also pointed to the constricting impact on practice of an emphasis on suicide risk and the associated formal structure of inquiries. By contrast, service users argued that consideration of risk, and especially new categories of risk, strengthened the control of clinicians over patients:

…they [the government] wanted to impose an extraordinarily centralist, dangerously severe personality disorder, DSPD – a completely new construction – onto psychiatric activity; that somehow you could detain someone merely because they happen to have potential personality characteristics that are going to possibly cause trouble in the future.^67^

More generally, other researchers have noted of the 2007 legislation that

‘anything goes’…[d]ecision-makers can draw relevant ingredients from (i) clinical and/or (ii) non-clinical factors existing in a patient’s diagnosis, characteristics, and/or circumstances…extensive scope for professional judgement within the [2007] legislation…[which] represents a significant threat to patient rights.^68^

The question remains as to whether recent attitudes to risk in mental health policy are different in kind, or just different in degree and in consequences, from earlier perceptions.^69^ In the regime of segregation and compulsory confinement that subsisted until the mid-twentieth century and beyond, it was taken as read that the risk of self-harm or violence was a reason for confinement, which could often induce families or communities to prefer the asylum in individual cases to other forms of care.^70^ Sarah York has observed that the avoidance of suicide risk was a major driver for regimes of restraint and surveillance in the Victorian asylum.^71^ However, as Åsa Jansson has recently shown, the significance of suicide, Lunacy Commission suicide statistics and asylum admissions in the Victorian period were far from straightforward, and owe as much to administrative intervention as clinical judgment^72^ – a tension also central in modern discussions of risk. Additionally, Harvey Gordon has noted that the specific risk of re-offending was a consideration in the discharge of criminal lunatics from Broadmoor.^73^ Yet the political and public discourse about mental illness in the nineteenth and early twentieth century did not emphasise risk as a reason for change (or continuity) in policy. Occasional newspaper excitements were deflected by leading psychiatrists who on one occasion in 1896 observed that ‘no alterations in the Lunacy Law will prevent …the occasional discharge of a patient who may subsequently become homicidal’ and called sarcastically for ‘another Act of Parliament providing that all persons who are likely to become homicidal shall present themselves periodically at the nearest lunatic asylum’.^74^ *Plus ça change*; but on this occasion, and for nearly a century thereafter, the moral panic which they were challenging was not translated into policy.

### 4.3 Resources

Much of the substance of any public policy lies in decisions about the allocation of resources. Seminar members were greatly exercised by the cost of services, but discussion threw up as many questions as answers. A starting point was that for at least a century before the inauguration of the post-war welfare state, mental health was one of the better-funded public welfare services, with large capital investments in asylums and a national regulatory system in the Board of Control.^75^ In recent memory, though, services have been regarded as underfunded because NHS expenditure on physical medicine has grown very much faster than expenditure on mental health, and because definitions of need (and thus of unfulfilled need) have continuously evolved. Witnesses were able to point out that NHS expenditure had normally not kept pace with changes in need (except, briefly, in the increased spending of the last Labour government), that it continued to fall behind growth in expenditure on physical medicine, and that within the mental health budget there have been contestable allocations with rapid recent increases (as noted previously) in spending on forensic and secure services, an emphasis on services for adults of working age at the expense of children and older people, and persistent large variations between regions. The failure to recognise the resource demands of community-based care, from the very beginning of the de-institutionalisation programme, was taken as read. It was noted that ministers had demanded, and got, evidence of efficacy in minute detail before agreeing to fund, and then continue funding, the IAPT programme.^76^

Given this widespread preoccupation with cost and resource constraints, historians will find it necessary, but difficult, to go behind these words and selected indicative statistics to build a balanced and informative picture of changes over time in actual resource allocation and the policies which drove it. The first attempt to synthesise data on the totality of NHS expenditure on mental health was only made in 2001/2, and at first covered only adults of working age.^77^ Before that time, information on NHS expenditure existed in the reports of the Department of Health and its predecessors, but this was not collected on a consistent basis before 1974 and did not reliably separate mental health from other expenditure; and within mental health it did not necessarily report expenditure within categories which are meaningful for policy analysis. Even after 1974 ‘changes in accounting procedures mean that no meaningful comparison of spending over the period can be made’.^78^ A more rigorous account of the total costs of mental health provision would require an assessment of all the agencies and institutions which have provided care and treatment; and in the differentiated environment which has emerged since the 1980s this would include local authorities, third sector and private providers, and increasingly the private funding of new forms of therapy such as counselling and of long-term residential care for dementia sufferers. Local authority expenditure is published in annual departmental reports but does not separate mental health from other social services expenditure after the establishment of generic social service teams. Where data from third sector and private providers is available it does not reliably distinguish mental health from other forms of social support. An even more elusive and fluid problem is the resourcing of care for people with dementia. Successive acts of policy have moved patients away from NHS institutions and towards local authority provision or private or third sector institutions. As the number of patients in long-term residential care, or requiring care in the community, has increased, the share of the cost borne by public expenditure has dropped.^79^ While this has prompted passionate controversy over many years about the principles of public funding for care, no consistent distinction has been made in that debate between mental and physical reasons for dependency and ‘meaningful comparisons’ are consequently difficult. On resource issues, as in many other perspectives on the mental health services, fragmentation of provision has led to fragmentation of evidence.

### 4.4 Critiques of psychiatry

Historians will also ask how the debate on the validity of psychiatric knowledge and practice – fascinating as it is to cultural historians – has been relevant to the development of services. In the 1970s, contestation of psychiatric theory and mental health practices came to be subsumed under the term ‘anti-psychiatry’, coined by David Cooper in 1971.^80^ Witnesses looking back from 2011 differed emphatically about the relationship between classical ‘anti-psychiatry’ and the range of critical positions which have since been taken about psychiatric practice. Anti-psychiatry of the 1970s included three very distinct strands, represented totemically by R.D. Laing, Thomas Szasz and Erving Goffman. Laing, influenced by existentialism and psycho-analysis, saw schizophrenia as a sane response to an insane environment created, largely, by dysfunctional families and capitalism. Szasz regarded mental illness as a construct misappropriating medical concepts in order to control people whose behaviour was regarded as alarming or offensive.^81^ Goffman regarded the asylum as a special case of the ‘total institution’, a closed society which manipulated its members into pathological behaviours,^82^ a perspective anticipated more pragmatically in the United Kingdom by Russell Barton.^83^ Questions raised, and answered very differently by seminar participants, include whether ‘anti-psychiatry’ had any influence beyond a brief flare-up, and what relation this alleged movement has to contemporary critiques of psychiatry and mental health. Colin Jones has argued that the term has included serious content-based critique of psychiatry, a ‘gestural politics of carnivalesque inversion and symbolic performance’, and the exploration of new paradigms of knowledge about what it is to be human.^84^

At the time, British psychiatric commentators were ambivalent about Laing and generally hostile to Szasz. Writers in *British Journal of Psychiatry* were inclined to treat Laing’s work ‘not as contributions to science or philosophy…but as part of the contemporary literature of social protest’,^85^ which allowed them to respect the verve and immediacy of his observations while deprecating the value of his analyses. But Kathleen Jones, a non-psychiatrist delivering the Maudsley Lecture in 1978, remarked that ‘behind the rhetorical excesses and the studied irrationality there are some serious points for psychiatry to consider’, in particular that ‘the patient’s view of what is happening to him is as valid as that of the therapist, and therapists ought to listen as well as to prescribe’.^86^ By the time of Laing’s death in 1989 this position was uncontroversial and commentators were observing that many psychiatrists had since entered the profession aspiring to an empathetic bond with their patients and that Laing’s contributions were ‘in the main welcome’.^87^ By contrast, Szasz was excoriated for ‘flashing rays of darkness on the whole field of psychiatry’ with an ‘utter inability to support his belief in [schizophrenia’s] non-existence with a shred of relevant…evidence’,^88^ and he was a principal, though not the only, target of a sustained rebuttal of challenges to the notion of mental illness as disease.^89^ Goffman got away lightly as a largely atheoretical observer whose ‘observations are so rich and his imagination so fertile that his books could supply social scientists with hypotheses for another generation to come’.^90^

In the seminars, one view, from senior psychiatrists, was that ‘the most striking thing’ about classical anti-psychiatry was that ‘eventually it disappeared, with practically hardly a trace left behind’ and that it all came to an end when ‘Ronny drank himself to death’.^91^ Another psychiatrist located its influence on front-line practice rather than psychiatric theory:

…the notion of mental illness or mental health was decried and denied by your average social worker who saw this all as social construction, capitalism, call it what you will…. And even as you’re standing on the corner of a Hackney street with a social worker going in to try and extract someone from a stinking house where they’ve been screaming and yelling for days and days, and neglect, pain, fear, and hallucinosis, you were still being told by your social worker friend, colleague, whatever, “Well, they’re not sure they’re going to let this person come into hospital; they’re not sure they’re really ill” because of their own political beliefs as opposed to any kind of medical or clinical understanding.^92^

Others identified the continuing relevance of anti-psychiatry to contemporary debate, whether in the narrow sense of a Laingian influence on ‘the rich tapestry of the psycho-analytic world’,^93^ or in a broader Foucauldian sense that ‘the whole of modern psychiatry is permeated by anti-psychiatry’^94^ in the form of contestation over the use of psychiatric diagnosis as an instrument of power. Witnesses noted for example that ‘…we’re still having to make an argument, relatively recently, to our own profession [clinical psychology] that actually we should abandon diagnosis, and replace it by formulation.’^95^

Several witnesses posited a relationship between critical psychiatry and the service user movement, in the sense that ‘individual and collective action by mental health survivors’ has done ‘a great deal to challenge the designations and treatments that have been foisted on people with mental health problems.’^96^ The link between the movement and classical anti-psychiatry has been challenged by others, and it has been argued that in the anti-psychiatric rhetoric of cutting loose from the system and challenging its legitimacy there had in fact been little engagement with the real problems of modern mental health care.^97^ It was noted, though, that the National Schizophrenia Fellowship of the 1970s was

…very much in the wake…of the anti-psychiatry culture as they perceived it. They thought that they weren’t being listened to, indeed that they were being perceived as pathological, and pathogenic, by psychiatrists, that they weren’t taken seriously. And their demand was to be put back on the map as regular, decent citizens.^98^

Historians of mental health services engaging with the history of psychiatric science will also need to address more recent and more eclectic critiques of psychiatry, connecting traditional anti-psychiatry challenges to the intellectual (and hence political) authority of psychiatric theory with new widely varying scientific and philosophical contributions to that theory, such as Thomas and Bracken’s attack on ‘bioreductionism’^99^, Fulford’s promotion of a values-based psychiatry,^100^ Moncrieff’s critique of the scientific basis of drug treatment,^101^ or Bentall’s deconstruction of the conceptual basis of diagnosis.^102^ Of the issues most relevant to practice and the delivery of services, witnesses emphasised the growing importance of neuroscience (incorporating behavioural genetics as well as more traditional psychopharmacology) in research and clinical practice. This has been the subject of intense debate within psychiatry, beginning with Craddock’s 2008 ‘Wake-up call’ in the *British Journal of Psychiatry*, which argued that although ‘evidence-based psychological and social interventions are extremely important in managing psychiatric illness’ they had resulted in a turning away from neuroscience which threatened the ‘downgrading of medical aspects of care’ and a ‘creeping devaluation of medicine’ in psychiatry.^103^ More recently, Bracken has argued in opposition to that view that:

Psychiatry now faces two challenges it cannot ignore. First, a growing body of empirical evidence points to the primary importance of the non-technical aspects of mental healthcare …second, real collaboration with the service user movement can only happen when psychiatry is ready to move beyond the primacy of the technical paradigm…Substantive progress in our field will not come from neuroscience and pharmaceuticals (important as these might be) but from a fundamental re-examination of what mental healthcare is all about and a rethinking of how genuine knowledge and expertise can be developed in the field of mental health.^104^

Within and beyond psychiatry, witnesses also emphasised the continuing controversy over the application of the paradigms of evidence-based medicine to mental health problems – a paradigm which was perhaps most prominent in the introduction of psychological therapies through IAPT, but one which discomfits many clinicians whose favoured therapies do not fit easily into randomised controlled trials.^105^ NICE guidelines, overtly based on research evidence, have been the principal engine of change in psychiatric practice in Britain in the last decade, but historians are bound to ask whether the conclusions drawn from evidence have been influenced by competing professional interests as well as by evidence, and to seek to measure how far the guidelines have in fact been absorbed into practice.

Underlying this discussion, and especially important for the historiography of mental health services, is the changing understanding of the definition of mental health or the scope of mental illness, and thus the ‘need’ for services. The willingness of many service users (and many non-users) to challenge professional definitions of their distress as illness is an evident legacy of the anti-psychiatry movement, but debate about scope and definition has been just as intense within and between communities of service providers. Psychiatric diagnosis is denounced as an instrument of power by critics of the disease model of mental distress, and the major diagnostic schemas, DSM-IV and ICD-10, are attacked by critics because, *inter alia*, they have been seen to include more and more people in stigmatised categories, especially by defining behaviour disorders as illness. On the other hand, the idea that mental illness is definable and treatable as a disease process like any other has been fundamental to many developments in service delivery in the period covered by these seminars. Diagnosis is hardwired into the logic of NICE guidelines.^106^ Understanding service development therefore depends on an historical understanding of the debate between formulation and diagnosis, and of the evolution of dimensional models of mental disorder; and these discussions extend far beyond the ideological struggle around classical anti-psychiatry.

### 4.5 Professions and values

To the outsider the mental health professions appear notably tribal, even to the extent of identifying specific positions within clinical and scientific debates with loyalty to specific professional communities.^107^ The Hackney street corner anecdote recounted above could be read as a vignette of inter-professional rivalry as much as a philosophical disagreement, and the same could be possibly be said of Craddock’s ‘Wake-up call’. Witnesses acknowledged that such tribalism existed, and associated it particularly with the separateness of training regimes, but raised further questions which clearly demand historical investigation. The orderly hierarchy of mental health professions which characterised the 1950s has been gradually replaced by an uneasy structure in which psychiatry maintains its supremacy with some discomfort (while still maintaining a separate pay scale not subject to inter-professional comparisons); mental health nursing has developed an autonomy and range of functions, especially those of community treatment, quite unknown to the nurses and attendants of the 1950s; clinical psychology has become established as a key agent in treatment of many disorders; a variety of other therapy professions has been created, some by policy and some organically, with distinct value systems and skill sets. Reports of the Department of Health’s *New Ways of Working* project from 2004 describe, perhaps optimistically, how a different style of leadership and clinical responsibility shared between different professions has come to exist in NHS mental health services.^108^ At the same time, front-line workers in the IAPT programme and support workers in recovery programmes generally work within their respective sets of treatment protocols but do not have specific professional allegiances.

The sharing of knowledge, skills and values between professional groups is of particular interest. In 2004, Thornicroft described a ‘third period’ of service delivery, following the rise and subsequent decline of the asylum, in which:

…community-based and hospital-based services commonly aim to provide treatment and care that are close to home, including acute hospital-care and long-term residential facilities in the community; respond to disabilities as well as to symptoms; are able to offer treatment and care specific to the diagnosis and needs of each individual; are consistent with international conventions on human rights; are related to the priorities of service users themselves; *are coordinated between mental health professions and agencies*; [our italics] and are mobile rather than static.^109^

The post-1959 services described by our participants aspired to all of those things, though there is room for profound scepticism about how far any of them were achieved. In particular, the delivery of community-based services depended on the relations between specialist services and GPs on the one hand, and between doctors (and psychologists) and nurses on the other. There has over the years been some research into the acculturation of GPs into a psychiatric mode of thought;^110^ given the level of GP activity in mild to moderate illness, it will be important to track how this has changed over time, in intention and effect. In the absence of GP representation at the seminars, participants had little to say about it. More was said about the transfer of skills to nurses and to other types of therapist under IAPT, but it is important to recognise that this sort of skills transfer long predated the twenty-first century: the Maudsley hospital started to train nurse-therapists in psychological therapies in 1972,^111^ and the expanded role of Community Psychiatric Nurses has depended on the acquisition of skills formerly confined to psychiatrists and psychologists.^112^

Historians need to account for the development of value systems within each of these professional communities and programme groupings and for the changing patterns of interactions between professional groups, exploring not only the training regimes but also the patterns of recruitment into different professions (and hence the social distance between different professions and between professionals and their clients) and the structures supporting professional identity, such as professional associations and interest groups. Witnesses were clear that some professions were originally only open to those who could afford to pay for training,^113^ while others have had a skewed ethnic or gender balance.^114^ Intellectual developments outside the clinical sphere, from behavioural psychology to genetics, have been transmitted through the lens of university training and acculturation into the clinical practice of different professions. The adoption of new professions into NHS training systems and into regulatory structures such as (most recently) the Health and Care Professions Council on the one hand validates the professions, yet on the other serves to constrain their autonomy and arguably distort their identity – hence the movement among some psychotherapists to resist regulation by the HCPC.^115^ Histories of professional groups, thus informed, will complement histories of mental health practice which focus on the distinctive contributions of individual psychiatrists or other professionals, thus providing an alternative ‘history of psychiatry’.

## 5 Towards a history of modern mental health services

Where should all this lead us? We began by remarking that a traditional historical narrative structured around rights is now enriched by new organising categories such as costs, risks, needs and values such as the aspiration to equality and inclusion. The seminars allowed an effective exploration of these issues, if only within the very real epistemological constraints of elite oral history. That said, they confirmed that the subject of the historical narrative has expanded and changed. Historians of the period up to the mid-twentieth century have rightly and fruitfully concentrated on madness and its management, exploring principally the experience of those identified (and mostly legally defined) as lunatics and the social, cultural, political, medical and institutional context of their treatment.^116^ Such a scope, valuable and innovative though it has often been, cannot serve the history of a period as complex as the one covered in these seminars. The history of modern services, for example, will necessarily include the experience of all service users, including a majority who never enter an institution and whose mental distress may well be entwined with various social and economic disadvantages whose causes and consequences are also part of the story.^117^ The voice of the service user will be represented not only as a set of individual narratives but also as a social and political movement, part of civil society, and an important strand in the history of clinical practice. Further, the history of mental health services will not make sense unless it is set within a broader analysis of the health economy and the organisational development of the NHS, of social services within local authorities, of the charity sector and of prisons, all of which deliver services to these users. We would expect a history of the professions to have more a sociological than an institutional character, exploring the evolution of value systems as well as patterns of recruitment and training; on the other hand, the political origins of state regulation and its consequences for the development of professions and their practices should be explored without preconception, Foucauldian or otherwise. In studying the politics of mental health policy, we would look to the significance of moral panic about risk in driving political discourse, but also to the lower-level interaction between officials and expert and lay networks in shaping policy and legislation, whether this was about resource allocation or civil liberties. Our history of psychiatric science would engage with classic anti-psychiatry and the critiques of psychiatry by psychiatrists, but also examine the impact of developments in psychology and neuroscience and the social and institutional structures in which research is done and disseminated into clinical practice.

Reflection on the fragmentation of mental health services since 1959 leads us to further, sometimes uncomfortable, speculation about the opportunities and risks in the historiography of earlier periods. Acknowledging, as our witnesses would lead us to believe, that the NHS in England and Wales had not by 1959 created a coherent system out of the people and institutions which it inherited on its establishment, should we not also regard the great asylums of the ‘water tower’ period, and the psychiatry practised within them, as rather less a monolithic and inclusive system for the care or control of deviants than a part of a much larger range of mixed institutions and contexts in which the ‘service users’ of the time had to live out their lives? The notion that the users of mental health services have more in common with other disadvantaged categories (the physically ill, the poor, ethnic minorities, immigrants) than they have special features is a powerful heuristic, widely discussed for the nineteenth and early twentieth centuries,^118^ and it could take us usefully beyond the conceptualisation of such categories as ‘deviant’. Just as we ask, of the modern period, how and why (and to what extent) certain modes of treatment are incorporated in practice and others dropped, should we move beyond the classic studies of developing psychiatric thought in the works of great men to a search for explanations of the practice and scientific beliefs of the front-line asylum doctors and general practitioners who delivered ‘care’ in the nineteenth and early twentieth centuries? Could the copious records of the Victorian and Edwardian civil service yield a more nuanced account of the interaction of laymen, professionals, politicians and officials in the ‘triumph of legalism’ in the 1890 Lunacy Act^119^ or the more contestable triumph of eugenic thought in the 1913 Mental Deficiency Act, and the institutional changes which followed those pieces of legislation?

We acknowledge that this is not a comfortable prospectus, whether for the modern or the more remote period. The very concept of mental health and mental illness has been enlarged and transformed in the last half-century; mental health policy has become confounded with many other aspects of public policy and mental health services have consequently grown and fragmented. Interaction between the fragments makes for a complicated story, but the alternative is a ‘single-issue mythology’ which will mislead rather than enlighten.

## Footnotes

1.
Volker Hess and Benoît Majerus, ‘Writing the History of Psychiatry in the 20th Century’, *History of Psychiatry*, 22, 2 (2011), 139–45; see also Greg Eghigian, ‘Deinstitutionalizing the History of Contemporary Psychiatry’, *idem*, 201–14; Eric Engstrom, ‘History of Psychiatry and its Institutions’, *Current Opinion in Psychiatry*, 25, 6 (2012), 486–91.

2.
Paul Hoff, ‘Die psychopathologische Perspektive’, in M. Bormuth and U. Wiesing (eds), *Ethische Aspekte der Forschung in Psychiatrie und Psychotherapie* (Cologne: Deutscher Ärzte-Verlag, 2005), 71–9.

3.
Contributors were drawn exclusively from England, and the cultural and institutional differences of Scotland, Wales and Northern Ireland are consequently not addressed in this paper.

4.
See Appendix for a list of speakers.

5.
See A. Seldon and J. Papworth, *By Word of Mouth – ‘Elite’ Oral History* (London: Methuen, 1983) and Lynn Abrams, *Oral History Theory* (London: Routledge, 2010). Most methodological discussion of elite oral history concentrates rightly on the limitations of memory, the need for triangulation between witnesses and with contemporary documents, and the pitfalls of a narrative co-constructed as a conversation between historian and witness (on which see in particular E.M. McMahan, *Elite Oral History Discourse: a Study of Cooperation and Coherence* (Tuscaloosa: University of Alabama Press, 1989)). These issues are of particular salience when the objective is to produce the most accurate possible account of a sequence of events. Our project was to generate themes for enquiry, and in this case the challenge was more to situate the interpretations of contributors in existing literature. A more forensic and less discursive approach will be necessary if, in a subsequent study, contributors are to be asked to provide evidence for answers to the questions which might be posed in such enquiries.

6.
Cf. J.K. Wing and J.M. Hailey, *Evaluating a Community Psychiatric Service: The Camberwell Register 1964–71* (Oxford: OUP, 1972); D.H. Bennett and H.L. Freeman, *Community Psychiatry* (London: Churchill Livingstone, 1991).

7.
Policy aspirations are encapsulated in two documents: Department of Health and Social Security, *Hospital Services for the Mentally Ill* (London: HMSO, 1971); *Better Services for the Mentally Ill*, Cmnd 6233 (London: HMSO, 1975).

8.
The disclosures of ill-treatment and malpractice in *Report of the Committee of Inquiry into Allegations of Ill-Treatment of Patients and other irregularities at the Ely Hospital, Cardiff*, Cmnd 3975 (London: HMSO, 1969) were followed by 17 further enquiries in the subsequent decade of which the most notable related to Whittingham Hospital (1972), Napsbury Hospital (1973), South Ockendon Hospital (1974) and Normansfield Hospital (1978).

9.

Nikolas Rose , ‘Psychiatry as Political Science: Advanced Liberalism and the Administration of Risk’, History of the Human Sciences, 9, 2 (1996), 1–23.

10.

David Healy , ‘Service Utilization in 1896 and 1996: Morbidity and Mortality Data from North Wales’, History of Psychiatry, 16, 27 (2005), 27–41.1598136410.1177/0957154X05044604

11.

Tom Burns , Community Mental Health Teams: A Guide to Current Practices (Oxford: Oxford University Press, 2004).

12.
Department of Health Circular HC(90)23/LASSL(90)11 (London, 1990). The CPA was simplified in 1999 as part of the Labour government’s reforms: Department of Health, *Effective Care Co-ordination in Mental Health Services* (London, 1999).

13.

D. Clark , ‘Improving Access to Psychological Therapy: Initial Evaluation of Two UK Demonstration Sites’, Behaviour Research and Therapy, 47, 8 (2009), 637–728.1964723010.1016/j.brat.2009.07.010PMC3111658

14.
M. Shepherd *et al.*, ‘Minor Mental Illness in London: Some Aspects of a General Practice Survey’, *BMJ* (1964), 2, 1359–63, amplified in Michael Shepherd *et al.*, *Psychiatric Illness in General Practice* (London: Oxford University Press, 1966).10.1136/bmj.2.5421.1359PMC181730714208202

15.
David Goldberg, ‘Epidemiology of Mental Disorders in Primary Care Settings’, *Epidemiologic Reviews*, 17, 1 (1995), 182–90. The three-nation survey was built upon a model set out in David Goldberg and Peter Huxley, *Mental Illness in the Community: The Pathway to Psychiatric Care* (London: Tavistock, 1980).10.1093/oxfordjournals.epirev.a0361748521936

16.
T. O’Sullivan *et al.*, ‘Goldberg and Huxley’s Model Revisited’, *Psychiatric Bulletin*, 29 (2005), 116; T. O’Sullivan *et al.*, ‘Goldberg and Huxley’s Model 27 Years On’, *Psychiatric Bulletin*, 31 (2007), 316.

17.
David Goldberg, Transcript, 17 December 2010, 30.

18.
Hugh Freeman, Transcript, 17 December 2010, 9.

19.
Goldberg, *op. cit.* (note 17), 3.

20.
John Hall, Transcript, 17 January 2011, 3.

21.
Elaine Murphy, Transcript, 7 April 2011, 2.

22.
Diana Rose, Transcript, 3 December 2010, 3.

23.
Trevor Turner, Transcript, 31 January 2011, 9.

24.
Hall, *op. cit.* (note 20), 26, citing in particular the work of Wolfensberger. See W. Wolfensberger, ‘Social Role Valorization: A Proposed New Term For the Principle of Normalization’, *Mental Retardation*, 21, 6 (1983), 234–9.6672533

25.
Freeman, *op. cit.* (note 18), 8.

26.
Goldberg, *op. cit.* (note 17), 5.

27.
Roy Griffiths, ‘NHS Management Inquiry’, Letter to the Secretary of State (London: DHSS, October 1983), reprinted in *BMJ*, 287, 6402 (1983), 1391–4; Martin Gorsky (ed.), *The Griffiths NHS Management Inquiry: Its Origins, Nature and Impact* (London: LSHTM, 2010).

28.
Roy Griffiths, *Community Care Agenda for Action: A Report to the Secretary of State for Social Services* (London: HMSO, 1988).

29.
Goldberg, *op. cit.* (note 17), 4.

30.
Hugh Freeman, Transcript, 31 January 2011, 17. On the role of front-line workers in policy making see, e.g., Michael Lipsky, *Street Level Bureaucracy* (New York: Russell Sage, 1980) as re-interpreted in Catherine Durose, ‘Revisiting Lipsky: Front-Line Work in UK Local Governance’, *Political Studies*, 59, 4 (2011), 978–95.

31.
Freeman, *op. cit.* (note 18), 9.

32.
Goldberg, *op. cit.* (note 17), 6.

33.
Turner, *op. cit.* (note 23), 11–12. In fact the Care Programme Approach, a set of management procedures for assessment, co-ordination and review, was developed by the Department of Health well before the Zito episode, as part of the implementation of the National Health and Community Care Act.

34.
Murphy, *op. cit.* (note 21), 3. On the other hand, the report by the National Confidential Inquiry into Suicide by People with Mental Illness (NCISH), *Patient Suicide: the Impact of Service Changes* (Manchester: University of Manchester, 2013) notes on page 10 that by 2011 ‘Trusts that had retained their specialist teams saw a larger fall in patient suicide than trusts that merged them [into generalist Community Mental Health teams]’.

35.
Hall, *op. cit.* (note 20), 4.

36.
Jim Symington, Transcript, 31 January 2011, 13. The phrase ‘ordinary life’ resonates with the title of a seminal King’s Fund paper, and its use, like the reference to Wolfensberger’s work above, illustrates the extent to which approaches originating in the care of people with learning disabilities generalised to other mental health services. King’s Fund, *An Ordinary Life: Comprehensive Locally-based Residential Services for Mentally-handicapped People* (London: King’s Fund, 1980).

37.
Symington, *ibid.*, 13.

38.
Hall, *op. cit.* (note 20), 4–5.

39.
Murphy, *op. cit.* (note 21), 2.

40.
Peter Bartlett, Transcript, 31 January 2011, 6.

41.
Peter Barham, Transcript, 3 December 2010, 8, quoting the words of a user group in 1990.

42.
Symington, *op. cit.* (note 36), 14.

43.
Department of Health, *The Mental Health Policy Implementation Guide* (London: HMSO, 2001).

44.
Louis Appleby, Transcript, 21 June 2011, 11.

45.
The evolution of the NSF is described by the chairman of the External Reference Group in G. Thornicroft, ‘National Service Framework for Mental Health’, *The Psychiatrist*, 24 (2000), 203–6.

46.
Rose, *op. cit.* (note 22), 15, in respect of the Recovery movement.

47.
Appleby, *op. cit.* (note 44), 11. The deployment of an economic case for treating mild mental illness can be traced back at least as far as 1957, in Sir Frederick Armer [Chairman of the Board of Control], ‘The Magnitude and Cost of Mental Illness’, *The Lancet*, 269, 1977 (1957), 1031.

48.
Rose, *op. cit.* (note 22), 9; Victor Adebowale, Transcript, 9 May 2011, 13–14.

49.
Goldberg, *op. cit.* (note 17), 5.

50.
David Pilgrim, Transcript, 17 January 2011, 8.

51.
Bartlett, *op. cit.* (note 40), 5.

52.
It is significant, at least culturally, that the first reports of the Ministry of Health after the establishment of the NHS were proud to report that over 97% of mental patients were being treated in hospitals accountable to the Ministry.

53.
See, eg., Roy Porter, *Madmen: A Social History of Madhouses, Mad-Doctors and Lunatics* (Stroud: NPI Media Group, 2004); Dale Peterson (ed.), *A Mad People’s History of Madness* (Pittsburgh: University of Pittsburgh Press, 1982); Nick Hervey, ‘Advocacy or Folly: The Alleged Lunatics Friend Society, 1845–63’, *Medical History*, 30, 3 (1986), 245–75.10.1017/s0025727300045701PMC11396503523075

54.
On the links to the 1980s see Peter Campbell, ‘From little acorns: the mental health service user movement’, in Sainsbury Centre for Mental Health, *Beyond the Water Towers: The Unfinished Revolution in Mental Health* (London: Sainsbury Centre for Mental Health, 2005), 73–82. On these groups, see also Nick Crossley, *Contesting Psychiatry: Social Movements in Mental Health* (Abingdon: Routledge, 2006). Crossley contrasts these movements to an earlier generation of mental health movements led by clinicians, social workers and philanthropists which aimed to extend the reach of services in the interests of ‘mental hygiene’. See also Helen Spandler, *Asylum to Action: Paddington Day Hospital, Therapeutic Communities and Beyond* (London: Jessica Kingsley, 2006).

55.
Campbell, *ibid.*, 76.

56.
On present-centredness in historical interpretation, see Adrian Wilson and T.G. Ashplant, ‘Whig History and Present-Centred History’, *The Historical Journal*, 31, 1 (1988), 1–16.

57.
Rose, *op. cit.* (note 22), 5.

58.
Rose, *op. cit.* (note 22); Glenn Roberts and Paul Wolfson, ‘The Rediscovery of Recovery: Open to All’, *Advances in Psychiatric Treatment*, 10 (2004), 37–48, and from another perspective, Deborah Mountain and Premal J. Shah, ‘Recovery and the Medical Model’, *Advances in Psychiatric Treatment*, 14 (2008), 241–4; Joel Braslow, ‘The Manufacture of Recovery’, *Annual Review of Clinical Psychology*, 9 (March, 2013), 781–809; Nikolas Rose, ‘“Screen and Intervene”: Governing Risky Brains’, *History of the Human Sciences*, 23, 1 (2010), 79–105.10.1177/095269510935241520518155

59.
Ulrich Beck, *World Risk Society* (Cambridge: Polity Press, 1990); Robert Castel ‘From dangerousness to risk’ in G. Burchell, C. Gordon and P. Miller (eds), *The Foucault Effect: Studies in Governmentality* (Hemel Hempstead: Harvester Wheatsheaf, 1991); Niklas Luhmann, *Risk: a Sociological Theory* (New York: De Gruyter, 1993); Nikolas Rose, ‘Governing Risky Individuals: The Role of Psychiatry in New Regimes of Control’, *Psychiatry, Psychology and Law* 5, 1 (1998), 177–95.

60.
Rose, *op. cit.* (note 22), 4.

61.
Murphy, *op. cit.* (note 21), 16.

62.
Murphy, *op. cit.* (note 21), 4, 6.

63.
See, eg., HC Deb., 10 July 1961, 644, 26–7; HC Deb., 23 October 1961, 646, 548; ‘Medico-Legal – Detention for Forty Years’ *BMJ*, 2, 5250 (August 1961), 527; *The Times*, 30 July 1961.

64.

*Report of the Committee on Mentally Abnormal Offenders*, Cmnd 6244 (London: HMSO, 1975).

65.
Turner, *op. cit.* (note 23), 11, 12.

66.
Rose, *op. cit.* (note 59), 190; Castel *op. cit.* (note 59), 293, 288.

67.
Turner, *op. cit.* (note 23), 24.

68.
Nicola Glover-Thomas, ‘The Age of Risk: Risk Perception and Determination Following the Mental Health Act 2007’, *Medical Law Review*, 19, 4 (2011), 604.10.1093/medlaw/fwr02322038745

69.
We are obliged to Dr Tony Black for discussions of Victorian attitudes to risk.

70.
See, eg., Joseph Melling, Bill Forsythe and Richard Adair, ‘Families, communities and the legal regulation of lunacy in Victorian England: assessments of crime, violence and welfare in admissions to the Devon Asylum, 1845–1914’, in Peter Bartlett and David Wright, *Outside The Walls of the Asylum: The History of Care in the Community, 1750–2000* (London: Athlone Press, 1999), 153–80.

71.
Sarah Hayley York, ‘Suicide, Lunacy and the Asylum in Nineteenth Century England’, University of Birmingham PhD thesis, 2009, esp. 180–294.

72.
Åsa Jansson, ‘From Statistics to Diagnostics: Medical Certificates, Melancholia, and “Suicidal Propensities” in Victorian Psychiatry’, *Journal of Social History* 46, 3 (Spring 2013), 716–31.10.1093/jsh/shs120PMC384259324293701

73.
Harvey Gordon, *Broadmoor* (London: Psychology News Press, 2012), esp. chap. 2.

74.
Charles Mercier, reporting on recent medico-legal cases in *Journal of Mental Science*, 42 (1896), 230. Mercier was commenting breezily on reports which included a double murder, two other murders, a suicide and a number of assaults.

75.
Freeman, *op. cit.* (note 30), 18.

76.
David M. Clark, Transcript, 17 December 2010, 14.

77.
Department of Health, ‘The Findings of the Financial Mapping Exercise 2002’ http://www.dh.gov.uk/en/Publicationsandstatistics/Publications/PublicationsPolicyAndGuidance/DH_4123379 accessed 12 September 2012.

78.
Gavin Thompson, *NHS Expenditure in England* (London: House of Commons Library, 2009), SN/SG/724, 7.

79.
Martin Knapp and Martin Prince, *Dementia UK: The Full Report* (London: Alzheimer’s Society, 2007), esp. chap. 6. On public expenditure share, see, eg., Richard Humphries, *Social Care Funding and the NHS* (London: King’s Fund, 2011). On principles, see Commission on Funding of Care and Support, *Fairer Care Funding* (Dilnot Commission Report) July 2011.

80.

David Cooper , Psychiatry and Anti-Psychiatry (London: Paladin, 1971).

81.
Thomas Szasz, *The Myth of Mental Illness: Foundations of a Theory of Personal Conduct* (New York: Harper & Row, 1974) and 34 other books on similar themes.

82.

Erving Goffman , Asylums (New York: Doubleday Anchor, 1961).

83.

Russell Barton , Institutional Neurosis (Bristol: Wright & Sons, 1959).

84.
Colin Jones, ‘Raising the Anti: Jan Foudraine, Ronald Laing and Anti-Psychiatry’, in Marijke Gijswijt-Hofstra and Roy Porter (eds), *Cultures of Psychiatry and Mental Health Care in Postwar Britain and the Netherlands* (Amsterdam: Rodopi, 1999), 283–94.

85.

J.K. Wing , ‘Laing and Goffman: Self and Others’, British Journal of Psychiatry, 118 (1971), 360–361.

86.

Kathleen Jones , ‘Society Looks at the Psychiatrist’, British Journal of Psychiatry, 132 (1978), 321–332.34613610.1192/bjp.132.4.321

87.
Anthony David, ‘In Memory of RD Laing’, *Psychiatric Bulletin*, 13 (1989), 696–8; A.W. Clare, ‘R.D. Laing 1927–89: An Appreciation’, *Psychiatric Bulletin*, 14 (1990), 87–8.

88.
M. Roth, ‘Schizophrenia and the Theories of Thomas Szasz’, *British Journal of Psychiatry*, 129 (1976), 323 & 325, responding to Szasz, *Schizophrenia: The Sacred Symbol of Psychiatry* (Syracuse: Syracuse University Press, 1976).10.1192/bjp.129.4.317987825

89.

R.E. Kendell , ‘The Concept of Disease and Its Implications for Psychiatry’, British Journal of Psychiatry, 127 (1975), 305–315.118238410.1192/bjp.127.4.305

90.
Wing, *op. cit.* (note 85), 361.

91.
Freeman and Goldberg, Transcript, 17 December 2010, 27.

92.
Turner, *op. cit.* (note 23), 9.

93.
Barbara Taylor, Transcript, 17 December 2010, 28.

94.
Barham, *op. cit.* (note 41), 6, citing Foucault.

95.
Pilgrim, *op. cit.* (note 50), 9; Division of Clinical Psychology, *Clinical Psychology: The Core Purpose and Philosophy of the Profession* (Leicester: British Psychological Society, 2010), defines formulation as ‘…the summation and integration of the knowledge that is acquired by th[e] assessment process that may involve psychological, biological and systemic factors and procedures. The formulation will draw on psychological theory and research to provide a framework for describing a client’s problem or needs, how it developed and is being maintained…. This provides the foundation from which actions may derive… Psychological intervention, if considered appropriate, is based upon the formulation.’.

96.
Barham, *op. cit.* (note 41), 23.

97.
Andrew Roberts, a service user who has taken a leading role in developing a service user perspective on the history of the movement, is one who has criticised this line of argument. For insight on the service user perspective on their own history and for a timeline of developments and a wealth of material on this history: http://studymore.org.uk/mpu.htm.

98.
Barham, *op. cit.* (note 41), 23. Mervat Nasser, ‘The Rise and Fall of Anti-Psychiatry’, *Psychiatric Bulletin*, 19 (1995), 743–6, sees MIND, transmuted from the World Federation for Mental Health in the UK in 1970, as embracing an anti-psychiatric philosophy (745–6).

99.

P. Bracken and P. Thomas , Postpsychiatry: Mental Health in a Postmodern World (Oxford: Oxford University Press, 2006).

100.

K.W.M Fulford , Moral Theory and Medical Practice (Cambridge: Cambridge University Press, 1989).

101.

Joanna Moncrieff , The Myth of the Chemical Cure (London: Palgrave, 2009).

102.
Richard Bentall, *Madness Explained* (London: Allen Lane, 2003); Richard Bentall, *Doctoring the Mind: Why Psychiatric Treatments Fail* (Harmondsworth: Penguin, 2010).

103.
Nick Craddock *et al.*, ‘Wake-up Call for British Psychiatry’, *British Journal of Psychiatry*, 193 (2008), 6–9. This was signed by 35 senior psychiatrists.10.1192/bjp.bp.108.05356118700211

104.
Pat Bracken, *et al.*, ‘Psychiatry beyond the current paradigm’, *British Journal of Psychiatry*, 201 (2012), 430–4. This was signed by 29 senior psychiatrists.10.1192/bjp.bp.112.10944723209088

105.
On the inapplicability of RCT, Margaret Rustin, Transcript, 17 January 2011, 12.

106.
Clark, *op. cit.* (note 76), 13.

107.
And see Benjamin Vicente *et al.*, ‘Attitudes of Professional Mental Health Workers to Psychiatry’, *International Journal of Social Psychiatry*’, 39, 2 (1993), 131–41.10.1177/0020764093039002068340213

108.
National Institute for Mental Health in England, *New Ways of Working for Everyone: Progress Report, April 2007* (London: Department of Health, 2007).

109.

Graham Thornicroft and Michele Tansella , ‘Components of a modern mental health service: a pragmatic balance of community and hospital care’, British Journal of Psychiatry, 185 (2004), 283–290.1545898710.1192/bjp.185.4.283

110.
See for example the second (1981) edition of Shepherd *et al.*, *Psychiatric Illness in General Practice* and D. Tantam and D. Goldberg, ‘Primary medical care’ in Douglas H. Bennett and Hugh L. Freeman, *Community Psychiatry: The Principles* (London: Churchill Livingstone, 1991), 361–85.

111.
See a good description in M.P. Bender, *Community Psychology* (London: Methuen, 1976), 38–45.

112.
See J.W. Rawlinson and A.C. Brown, ‘Community psychiatric nursing in Britain’, in Bennett and Freeman, *Community Psychiatry*, 463–87.

113.
Notably child psychotherapy at the Tavistock Clinic: Rustin, *op. cit.* (note 105), 10–11. This is still true for counselling psychology.

114.
Applications for clinical psychology training have been heavily skewed towards women, with an under-representation of ethnic minorities.

115.
See, eg., Pamela Austin *et al.* (34 signatories), ‘Regulation: Refusing to Participate’, *Therapy Today* 20, 6 (July 2009); Andrew Samuels, ‘HPC Regulation: More Harm Than Good’, *Therapy Today* 20, 5 (June 2009).

116.
For a partial exception, see Cathy Smith, ‘Family, Community and the Victorian Asylum: A Case Study of Northampton General Lunatic Asylum and its Pauper Lunatics’, *Family and Community History*, 9, 2 (2006), 109–24.

117.

Peter Huxley and Graham Thornicroft , ‘Social Inclusion, Social Quality and Mental Illness’, British Journal of Psychiatry, 182 (2003), 289–290.1266840110.1192/bjp.182.4.289

118.
See, eg., Peter Bartlett and David Wright, *Outside The Walls of the Asylum: The History of Care in the Community*, 1750–2000 (London: Athlone Press, 1999), which largely concentrates on the 19th century.

119.
The phrase was used by Kathleen Jones as a chapter heading in *Asylums and After*, and has provoked legal and medical historians ever since.

## Appendix

*List of Contributors*

**Victor Adebowale**, Chief Executive of *Turning Point* mental health charity and social enterprise.

**Louis Appleby**, Consultant Psychiatrist; National Clinical Director for Health and Criminal Justice, Department of Health, and Professor of Psychiatry at the University of Manchester.

**Peter Barham**, Psychologist; Honorary Fellow of the Wellcome Trust, author of *Closing the Asylum* (Penguin 1992, 1997).

**Peter Bartlett**, Nottinghamshire Healthcare NHS Trust Professor of Mental Health Law.

**David M. Clark**, Clinical Psychologist; Professor of Experimental Psychology, University of Oxford, formerly Professor of Psychology, Institute of Psychiatry.

**Hugh Freeman**, Consultant Psychiatrist; formerly Editor *British Journal of Psychiatry*.

**Sir David Goldberg**, Consultant Psychiatrist; Professor Emeritus, Institute of Psychiatry

**John Hall**, Clinical Psychologist; Professor at Oxford Brookes University.

**Elaine Murphy, Baroness Murphy**, Consultant Psychiatrist; formerly professor of Psychiatry at Guy’s Hospital Medical School, formerly Chair of NE London Strategic Health Authority.

**David Pilgrim**, Clinical Psychologist; Professor of Mental Health, University of Central Lancashire.

**Diana Rose**, Senior Lecturer in User-Led Research, Co-director Service User Research Enterprise (SURE), King’s College, London.

**Margaret Rustin**, Psychotherapist; formerly Head of Child Psychotherapy, Tavistock Clinic.

**Anthony Sheehan**, Mental Health Nurse; formerly Chief Executive, Leicestershire Partnership NHS Trust, formerly Director of Mental Health and subsequently Director-General, Health & Care Partnerships, Department of Health.

**Jim Symington**, Social Worker; formerly Deputy Director, National Mental Health Development Unit, Department of Health.

**Trevor Turner**, Consultant Psychiatrist; Clinical Director, City and Hackney Centre for Mental Health.

**Barbara Taylor**, Historian; Professor of Modern History at the University of East London.

